# Dynamical Constants and Time Universals: A First Step toward a Metrical Definition of Ordered and Abnormal Cognition

**DOI:** 10.3389/fpsyg.2017.00332

**Published:** 2017-03-07

**Authors:** Mark A. Elliott, Naomi du Bois

**Affiliations:** School of Psychology, National University of Ireland GalwayGalway, Ireland

**Keywords:** time quantum (TQ), taxonomic model of quantal timing (TQM), oscillation, cortical dynamics, abnormal cognition

## Abstract

From the point of view of the cognitive dynamicist the organization of brain circuitry into assemblies defined by their synchrony at particular (and precise) oscillation frequencies is important for the correct correlation of all independent cortical responses to the different aspects of a given complex thought or object. From the point of view of anyone operating complex mechanical systems, i.e., those comprising independent components that are required to interact precisely in time, it follows that the precise timing of such a system is essential – not only essential but measurable, and scalable. It must also be reliable over observations to bring about consistent behavior, whatever that behavior is. The catastrophic consequence of an absence of such precision, for instance that required to govern the interference engine in many automobiles, is indicative of how important timing is for the function of dynamical systems at all levels of operation. The dynamics and temporal considerations combined indicate that it is necessary to consider the operating characteristic of any dynamical, cognitive brain system in terms, superficially at least, of oscillation frequencies. These may, themselves, be forensic of an underlying time-related taxonomy. Currently there are only two sets of relevant and necessarily systematic observations in this field: one of these reports the precise dynamical structure of the perceptual systems engaged in dynamical binding across form and time; the second, derived both empirically from perceptual performance data, as well as obtained from theoretical models, demonstrates a timing taxonomy related to a fundamental operator referred to as the time quantum. In this contribution both sets of theory and observations are reviewed and compared for their predictive consistency. Conclusions about direct comparability are discussed for both theories of cognitive dynamics and time quantum models. Finally, a brief review of some experimental data measuring sensitivity to visual information presented to the visual blind field (blindsight), as well as from studies of temporal processing in autism and schizophrenia, indicates that an understanding of a precise and metrical dynamic structure may be very important for an operational understanding of perception as well as more general cognitive function in psychopathology.

## Dynamical Constants and Time Universals in Cognition

Successful cognition aims to provide as narrow a range of responses to a specific stimulus as possible and in so doing allow for contexts that are both immediate as well as those that are temporally disjunct. A good example of the latter is the ability to return service in tennis: In order to place the racket at precisely the correct position in space at the right time (and with the correct angular displacement and degree of force), the player has to – *a priori* – judge the future position of the tennis ball by calculating current velocity, which entails a spatiotemporal location prediction by means of run-time estimations. They may thus prepare their successful response on the basis of this information. In the spatial domain, a given stimulus may exhibit a wide variety of defining features that require assignment to their ‘object’ alongside segregation from the features defining surrounding stimuli. The problem to be solved by cognition is in both cases similar, except for the tennis player the ‘binding’ of different responses must occur over time to ensure that the eventual velocity code and response are veridical and successful. For successful figure-ground segmentation, there is not necessarily the same emphasis in coding over time (although maintaining identity irrespective of observer or object movement is also a factor for consideration). What is important in this instance is the correct assignment of features to objects. Of course cognition also includes the correct deployment of memory and use of language, and includes the mechanisms and processes responsible for the deployment of attentional resources. The discussions that follow may apply to any and all instances of cognition but this contribution will focus on those known to operate during perception.

The use of technologies such as the electroencephalogram (EEG) as well as the use of multi-unit single cell recordings, both of which allow for good temporal resolution in time-series data, has encouraged more widespread consideration of the dynamics of brain function as the operating characteristic of successful cognition. For instance, it is probably now widely held that the rhythmic phase synchronization of neural activity at frequencies in the EEG gamma band (i.e., 30–70 Hz) brings about the organization of visual events, in particular, the ‘binding’ of individual visual features to form perceptual wholes (Gray, 1999; Singer, 1999). Thus, the term binding refers both to ‘perceptual binding’ or the necessary relation between features that go together to form a wholistic, object-related representation (e.g., as defined in Treisman and Gelade, 1980), or the term refers to the mechanism by which neurons dedicated to responding to particular feature attributes (contrast orientation or direction of movement for example) combine to form assemblies. The two definitions are often treated as synonymous and theoretically, binding by synchronization is assumed to be isomorphic with the binding of feature attributes as introduced in our second example above. Although there is an argument that these definitions require careful reconsideration (see Elliott et al., 2006), this theory is now supported by a very considerable literature describing physiological studies.

## The Temporal Structure of Coding in Vision

The strengths of the physiological binding hypothesis lie in addressing a number of key theoretical problems: assembly coding is known to be necessary for neurons to code specific visual contrasts (oriented lines, edges, etc.) as opposed to simple changes in contrast, while the segregation of features defining figure from ground is achieved by virtue of neurons participating in different assemblies defined by a unique phase in their firing patterns. In this way features coding an object may be coded by virtue of oscillatory firing at one particular phase, while all features defining ‘ground’ or the context of the object may be responded to by firing at other phases of a given oscillatory firing regimen. This avoids what is referred to as a ‘superposition catastrophe’ when everything becomes coded in the context of a single assembly code, leading to there being no means of differentiating (for example) figure from ground.

Clear in these instances, and similar to the second example above, of returning a tennis serve, is that there needs to be a particular temporal code which permits two or more simultaneous assemblies to be active and not confusable. Also entailed is the need for this code to be precise, and almost certainly, regularly precise. Consider not only the coordination of perception and action, or the issue of differentiation of perceptual materials, if the brain functions as a machine, providing consistent output to match regularities in its input, it must also operate in the manner of a complex mechanical system, i.e., a system comprising independent components that are required to interact precisely in time. It follows that the precise timing of such a system is essential, not only essential but measurable, scalable (if the system has several interacting parts, each doing tasks of a different scale), and reliable over observations. The catastrophic consequence of an absence of such precision, for instance, that required to govern the interference engine in many automobiles, is indicative of how important timing is for the function of dynamical systems at all levels of operation. The implications of this for the timing of psychological processes in psychopathologies will be returned to later in this paper.

The question of precision seems difficult for physiological data for several reasons. First, studies of neural synchronization often do not report perfect phase alignment between oscillatory neuronal responses but may report separations in time of up to 20ms between the ‘synchronized’ responses of neurons in visual cortex (see e.g., Gray et al., 1989 for data and Singer, 1993 for review). This leads to approximation but not precision regarding the timing of the neural response. Second, and while techniques such as EEG allow a measure of the field potential, even if all other issues with the EEG were resolved, as regards timing precision, these measures are still of insufficient resolution to develop a precise model of the dynamics in the cognitive microstructures operating during recording. A third issue regards theory, which has from time to time concerned itself with the relationship between the timing of the neural response to the stimulus or other non-proximal event. A recent focus has involved discussion of the relationship between small eye movements (microsaccades) and induced cortical gamma activity, suggesting that recordings of cortical gamma-band activity are essentially related to the frequency of muscular movements in the eye (Yuval-Greenberg et al., 2008, see Hassler et al., 2011 for contradictory evidence). It is also suggested that a link exists between evoked-gamma (i.e., event-time locked) activity and visual grouping (see Tallon-Baudry, 2009; Martinovic and Busch, 2011 for reviews). This is controversial as other EEG studies, supported by a body of evidence in the physiological literature, generally holds oscillatory-gamma activity to be necessarily unrelated to the phase of stimulus events in order to allow for neurons to respond with a temporal code that is independent of stimulus events (see Pantev, 1995 as well as Tallon-Baudry and Bertrand, 1999; Fries et al., 2007, for reviews).

It is generally true to say that for the most part neurophysiology assumes a one-directional causal flow and so ignores psychological data. There are two sets of evidence though that indicate a very fine precision in the architecture of the psychophysical response, and at least one of these sets can be linked directly to neural synchronization via oscillation. The question addressed by this paper is of their commonality.

The two sets of data to be linked concern the taxonomic time-quantum model of Geissler (1985, 1992) and more particularly, the empirical support for this model provided by Geissler et al. (1999) and Geissler and Kompass (2001). The time-quantum model postulates (in principle all) successful cognition to depend upon interactions between processes according to a very precise temporal architecture based upon a fundamental time unit of around 4.5–4.6 ms. The first set of data derives from the studies described by Elliott and Müller (1998, 2000, 2004).

Elliott and Müller designed a paradigm in which the separate features that go together to form an object (in this instance the set of ‘L’ shaped corner junctions that go together to define a ‘square’ perceptual arrangement, hereafter referred to as a ‘target’) were primed by presentation of crosses presented simultaneously in square arrangement. Both priming crosses and target corner junctions were presented as one element of larger priming and target displays of crosses or corner junctions (respectively, see Elliott and Müller, 1998 for details). Priming displays (hereafter referred to as a ‘premask’ display) were dynamic in that crosses were divided over four successively presented image frames presented repeatedly and at frequencies in the EEG gamma band. The crosses presented in square arrangement (hereafter the prime) were presented as one of the four image frames and on half of all trials. The four priming crosses were therefore presented simultaneously. The premask display was presented for a period usually of 600 ms and was followed by a second display that on half of trials included a target. Elliott and Müller (1998, 2000) showed that priming stimulus presentation, below detection thresholds for premasks presented faster than an image frame presentation rate of 21 Hz, lead to faster detection reaction times (RTs) to a subsequently presented target than when the premask display did not contain a prime. The priming stimulus is defined as a prime because it was not found to cue target presentation.

Elliott and Müller (1998) identified a specificity in priming to premask displays that flickered at 40 Hz. Elliott and Müller (2004) subsequently introduced data from premasks presented at frequencies (in single Hertz steps) over the range 30–50 Hz. From these experiments, they concluded priming effects were not to be confined to 40 Hz, but instead occurred for premask matrices flickering at 33 Hz, 39–40 Hz, and at 46–47 Hz. Described in terms of a ‘Generalized Phase Angle Hypothesis’ (GPAH), Elliott and Müller (2004) observed that priming occurs for primes presented within premask rhythms that would all be in phase alignment at regular 148 ms intervals. This suggests modulation of the premask-presentation rhythm by a slower (EEG theta) rhythm estimated at that time to be 6.75 Hz, with which the premask-matrix frequencies would share a common phase angle. The GPAH predicts that, for a given priming frequency *f* and corresponding period duration *t* = 1/f, facilitation reoccurs at every time point.

$$\begin{array}{c} J(t)=t(nt+0.5)-T \end{array}$$

where *nt* is an integer multiplier of frequency and *T* denotes a quantal time delay constant. The term +0.5 accounts for the observation that, when f = 40 Hz, priming occurs optimally for targets presented at times equivalent to phase angles of 180° relative to the rhythm of premask-matrix presentation (see Elliott and Müller, 2000, 2004, for full explanations).

Prime generation under the circumstances in which regularly ordered stimulus frequencies interact in phase with a slower perhaps endogenous rhythm is only one part of the story. An analysis of the same dataset, published earlier by Kompass and Elliott (2001) found that priming varied in magnitude (or priming was or was-not present) for frequencies according to the time of target presentation expressed in terms of the phase of the premask-matrix presentation frequency (referred to in terms of a ‘Return Phase Hypothesis’ or RPH for oscillatory priming). In fact priming was maximal for targets presented at a time shortly ahead of the premask-matrix presentation phase at which the priming-stimulus would have been presented if premask-matrix presentation had continued. This indicates the prime to be a cognitive response that can develop *in advance* of the priming stimulus, most likely facilitated by the rhythmic nature of premask-matrix presentation. On the assumption that an advanced prime response, develops in ‘anticipation’ of target presentation, the prime may be considered ‘protentive’ in the sense used by Husserl et al. (1893–1917), to denote a psychological event that occurs in advance of, is related to, and persists into the period of time at which a subsequent psychological event occurs.

Elliott (2014) provided analyses of nine datasets covering priming frequencies across the range 27–68 Hz with the following conclusions: priming in the sense of anticipatory coding (as described by the RPH) occurs maximally when priming frequencies are in phase with a more precisely estimated 6.69 Hz rhythm, but not when priming frequencies and the slower rhythm are out-of-phase. When out of phase with the 6.69 Hz rhythm, targets are primed when presented at phases that are synchronous with or slightly lag priming-stimulus presentation phase. On this basis, Elliott concluded that an anticipatory coding of the target occurs when the oscillation imposed by the stimulus (or the oscillation at which stimulus-coding occurs spontaneously) is aligned in phase with the phase of the slower oscillation. Elliott also showed that this anticipatory code is not generated when this interaction in phase does not occur. It cannot be concluded that this interaction is particular to 6.75 Hz; mathematically several other frequencies may align phases at other times, which allows for other slow rhythms to be instrumental for anticipatory coding.

The second set of psychophysical evidence refers to a theory of quantal timing (TQM) originally advanced by Geissler (1985, 1992) and re-evaluated against empirical data by Geissler et al. (1999) and Geissler and Kompass (2001). Geissler et al. (1999) discovered a discrete structure in a set of preferred inter-stimulus intervals (ISIs) at 4.5, 9.5, 18, 22, 27, 37, 44, 54, 107, and 145 ms, for the shift from apparent motion between two spatially separated but independent and temporally asynchronous changes in brightness, to the perception of flicker between these changes. These intervals also exhibit close relation to a set of periods extracted from von Békésy’s (1936) data. von Békésy (1936) discovered a series of 11 local invariances in the absolute intensity threshold for sound as a function of frequency. In his experiment, frequency was increased continuously from below threshold until the stimulus became noticeable while intensity was kept constant. Local invariances were defined as points at which the stimulus becomes noticeable at the same frequency for different intensities. For instance, at 18 Hz the same point of transition was observed at six levels of intensity. Geissler and Kompass (2001) note that the observation of multiple distinct periods permits the extraction of further regularities. Of most importance are regularities in temporal spacing between periods for which there is a higher probability of perceptual change. Analysis reveals (with one exception) that these periods (reciprocals to the frequencies used by von Békésy) may be grouped into three subseries of approximate harmonic doublings: 222, 111, 55, and 26 ms; 133, 91, and 45.5 ms; 133, 72, and 36 ms.

Statistically, every member of these sets exhibits a close-to integer ratio to at least two other set members, indicating their structural relation in time. This structure is a fundamental tenet for Geissler’s (1985, 1992, 2004, 2009) and Geissler and Kompass (2001) taxonomic model of TQM. TQM is a hierarchical model of related temporal intervals derived initially from inspection of von Békésy’s data. The notion of a “time quantum,” derives from meta-analysis of these data, which suggests that each period is a multiple of a lower common divisor in the range 4.5–4.6 ms. Geissler postulates this value as representing an absolute quantum Q_0_, of which, multiples called operative quanta are linked. In the context of apparent motion, this architecture would permit a processing system to develop which possesses coherence across different scales; from the signaling of temporal discontinuous, but nonetheless simple, contrasts, to the mental representation of an interval.

Besides proposing Q_0_ (which corresponds to 220 Hz in the frequency domain), TQM differs from concepts of a central clock mechanism (e.g., Stroud, 1955) by virtue of the assumption of a limited coherence resulting from increasing loss of phase precision over time. This assumption is derived from the principle that mental timing obeys a form of Weber fractioning. TQM leads consequently to a conceptual overlay between quantal and Weber Laws – in which it is necessary to increase the operative quantum Q when an interval M ^∗^ Q is exceeded. This idea is supported by the results presented by Kristofferson (1980) who investigated discrimination of short intervals after prolonged training. He showed that such an overlay actually exists, while the estimate *M* == 30 for the concatenation of operative quanta is based upon Teghtsoonian’s (1971) finding that, on a subjective scale, the Weber fraction, often called Ekman’s Constant, is about ^1^/_30_.

A third basic assumption of TQM is the formation of hierarchies of entrained intervals. In other words, intervals are the periods of a given oscillation and the TQM is a model of interrelated oscillations. The idea of a hierarchy suggests that preferences should exist for multiples of the quantum, composed of common divisors of integers such as 24, which are inherent in the empirical data on discrete timing (Geissler, 1992; Kompass, 1999). The experiment on long-range Beta apparent motion (Geissler et al., 1999) is the first in which elementary TQM predictions were confirmed by experimental data. What are referred to by Geissler et al. (1999) as independent critical ISIs (cISI) are very close to multiples (1, 2, 4, 5, 6, 8, 10, 12, 24, and 32) of a period of 4.49 ms. A second empirical derivation of Q_0_ based on measures of local variation of ISI has given an estimate of 4.56 ms (Kompass, 1999). The set of all cISI spans the interval [0, 32 ^∗^ Q_0_] and this is in support of the hypothesis of limited coherence with *M* = 32.

Quantal timing, thus describes a psychophysical processing architecture based around operative quanta. These quanta are a set of different processing intervals that relate numerically through their multiplicative relations with a fundamental interval Q_0_. Operationally, operative quanta rescale due to the phase coherence of any given operative quantum persisting for no more than 32 or 33 cycles. After this the operative quantum rescales to another operative quantum in a manner empirically consistent with Weber fractioning. Different processing intervals associated with different quanta may serve different psychophysical or cognitive functions. It might be assumed that more complex cognition (associated, for example, with perception of apparent motion) is a concatenation of less complex cognitive events (e.g., a discrete sampling, at lower scale, of contrasts over time and space). Based upon this idea, as well as the idea, common with the physiological binding hypothesis, that neural oscillations are responsible for cognition, a ‘dynamic binding’ of the different process states is a fundamental concept within TQM.

This conceptualization is thus strongly suggestive of a relationship between TQM and the models arising from Elliott and Müller’s research in which the notion of a temporal binding is central. However statistically, this relationship requires the following correspondences: (i) establishing a constant link between a value of Q_0_ in the range 4.5–4.65 ms and the priming frequencies, which for protentive coding are 32.25–33.5 Hz; 38–40 Hz; 45.75–46.5 Hz; 53.25–53.75 Hz; 59–60.75 and 65.75–67.5 Hz; (ii) establishing, if not a hierarchical relationship, a relationship in which frequencies at different scales can be seen to interact to bring about a particular psychophysical effect and finally (iii), from this set of data establish a relationship pointing toward a limit cycle of 32 ^∗^ Q_0_.

The slow frequency with which prime frequencies interact to bring about protentive coding (6.69 Hz) is 33 cycles of a period of a hypothetical Q_0_ of 4.53 ms, an estimate very close to that made by Kompass (1999) and at a multiple which would suggest a point in time of maximum coherence for Q if it is assumed that 33 == 0 with respect to coherence strength of a cyclical system subject to systematic coherence decay over 32 cycles. This would appear to satisfy correspondence (iii), although this applies only in the case where the prime brings about protentive coding, as is evident when a Lomb-Scargle periodogram is applied to the data (this is a method for finding periodicity in irregularly sampled or sparse data. It is analogous to the more familiar Fourier Power Spectral Density used for detecting periodicity in regularly sampled data). The absence of a significant relationship between priming and a slower frequency when primes occur in synchrony with, or following the phase of priming-stimulus presentation (see Elliott, 2014 for a more detailed discussion) appears to preclude consideration of correspondence (iii) from all instances of priming. This also indicates that priming may occur without interaction across frequencies with the corollary that where correspondence (iii) applies correspondence (ii) may also apply (as in the case of protentive prime formation), but where it does not, correspondence (ii) cannot apply.

The correspondence of particular frequencies to TQM = 4.53 is less straightforward because of unknown error in the estimates of the frequencies themselves and TQM, and, as a direct consequence the value associated with 33 cycles of TQM. There is also an issue concerning which frequencies should be taken into consideration. Reference to Elliott (2014) shows that the magnitude of priming is non-uniformly distributed over small bands from which one might assume either the mode or the central moment to represent the largest measure of successful priming. This compares against the idea that interactions in frequency, in which, in a hierarchical system one type of process might transform into another and would do so at a point in time equivalent to the origin of the process. If priming were to be distributed non-uniformly over a single frequency domain this could indicate the zero-crossings, or the frequencies in between bands of priming to be important. Empirically this may be less likely given the interaction of priming frequencies occurs at 149 ms (so with the slow 6.69 Hz rhythm). Combining data from Elliott and Müller (2004) and Elliott (2014) and assuming the sets of priming frequencies center on 32.75 Hz; 39.5 Hz; 46.38 Hz; 53.39 Hz; 59.75 Hz and 66.75 Hz, we can immediately see loose correspondence with frequencies which have oscillation durations that are direct integer multiples of 4.53 ms (calculated _4.53^∗^N_ with the given integer multiple [*N*], 32.75 Hz ≡ 30.53 ms [_4.53^∗^7_]; 39.5 Hz ≡ 25.32 ms [_4.53^∗^6_]; 46.38 Hz ≡ 21.56 ms [_4.53^∗^5_]; 53.39 Hz ≡ 18.73 ms [_4.53^∗^4_]; and 66.75 Hz ≡ 14.98 ms [_4.53^∗^3_]). These correspondences occur with discrepancies (in the time domain) of 1.18 ms; 1.86 ms; 1.09 ms; 0.61 ms; and 1.39 ms. Although small relative to frequency, relative to TQM these oblige reconsideration of TQM with a standard error in the estimate of 0.52 ms giving an estimate of TQM ranging between 4.01 and 5.05 ms. It is difficult to know where error should be attributable, perhaps to both frequency and TQM estimates, but in spite of this, the convergence of these frequencies at, and interaction with a slow frequency of 6.69 Hz, the 33rd (or 0) multiple of TQM = 4.53 ms remains convincing.

## The Coding of Complex Sound: Structural Analogies with Vision

A similar auditory priming paradigm has also been used to examine the frequencies, and phasic interactions involved in the dynamic system of auditory binding. The original of this paradigm, designed by Aksentijevic et al. (2011), evokes an auditory gamma-band response (aGBR, i.e., a transient oscillatory brain response evoked by the onset of auditory stimuli in the range 30–70 Hz, see Pantev et al., 1991), which may be phase locked to the stimulus by means of stimulus entrainment (Aksentijevic et al., 2011, 2013, 2014), so essentially identical to that originally developed by Elliott and colleagues. Entrainment refers to the alignment in phase, or coupling of two independent oscillatory systems, in such a way that their periods of oscillation become related (Cummins, 2009) and in this case, the auditory stimulus evokes an aGBR that is phase locked to its frequency.

Results have consistently revealed expedited RTs to targets that comprise frequencies which are fractional multiples of the entrainment frequency (i.e., are inharmonically related) compared to those that are harmonically related. Importantly, in audition these effects appeared to be specific to an entrainment rate of 33 pps (Aksentijevic et al., 2011, 2013, 2014). This effect is naturally close to at least one frequency that primes visual binding mechanisms: the period of a 33 pps entrainment rate is 30.3 ms, which is close to seven times Q_0_ (4.53 × 7 = 31.71 ms). More recently a variation of this paradigm varied the position of the entrainer tone in the repeating four-tone sequence that predates target presentation, and so entrainer timing was manipulated in a similar way to that reported by Elliott (2014) and is evaluable relative to a 6.69 Hz rhythm. Thus far the data only covers a narrow range of frequencies. However, analogous effects have been revealed when the target was presented both in and out of phase with 6.69 Hz. The target was presented out of phase with a 6.69 Hz oscillation, in two separate experiments. In both cases response latencies decreased when the target was presented at the beginning of the four pip entrainer’s oscillatory trace. This suggests that primed targets presented out of phase with a 6.69 Hz rhythm result in responses that are not protentive as occurs in the visual domain (Elliott, 2014). The target was also presented, in three separate experiments, in phase with a 6.69 Hz rhythm, resulting in expedited responses ahead of the first pip in the entrainer’s oscillatory trace. This suggests that again, as in visual binding, when there is an interaction in phase with a 6.69 Hz theta rhythm and frequencies in the gamma-band range, an anticipatory response is revealed for primed targets.

## Time Constancy and Cognitive Abnormality

It is important to consider the implications of empirical derivations of such small quanta. Essentially, this question concerns the origin and nature of noise. This is particularly important if frequencies are claimed to be the signature of functional oscillatory activity in the brain. It is also important when, for instance, repeated measurements identify ranges of relevant frequencies, while the neighboring integer multiple of the quantum concerned lies within or proximal to the range, although, corresponding variation at the scale of the quantum leads to a significant lack of precision. There are two questions that arise from this. The first asks how important reference to the quantum is if deviations are not only evident but likely? The second asks, what constitutes a ‘systems failure’ if there are such deviations. The corollary of this question is at which level might corrections be made if there is some systematic failure to maintain coherence between the neurons in a dynamic assembly?

An answer to the first of these questions remains to be seen but is also open to empirical enquiry. However, TQM may ultimately be better described not in terms of absolute thresholds for mental events, derived from a precise, fractional relation between TQ_0_ and a given operational quantum but instead in terms of the higher probability of a given mental event (such as the perceptual switch from apparent motion to flicker), with increased probability determined by the increased precision of the fractional relation between TQ_0_ and the operative quantum.

With respects to the second question, an appropriate question to ask is ‘how is the individual managing to do anything (cognitive) at all?’ At the heart of this question lies the well-known observation that given some types of brain damage, commensurate cognitive impairment reduces in severity over time, and in some cases this involves recruitment of alternative neural circuits to those originally subserving the cognition concerned. An example of this reorganization is described by Foucher et al. (2005), who found anatomically different patterns of functional connectivity that define the ‘core’ areas of cortex responsible for coding lexical decision and memory retrieval in patients with schizophrenia, for whom the core refers to inter-hemispheric interaction across frontal regions of the brain. Accepting that frontal brain regions can serve as a form of generalized processing resource (Duncan and Owen, 2000), the question remains by virtue of which principle could activity within frontal regions of cortex come to mimic impaired damage in other regions of cortex. By virtue of adopting the same basic dynamic structure perhaps?

Interestingly in this context is the putative connection between the observed operational quantum at 55 ms and work on simultaneity thresholds, undertaken initially by Brecher (1932) and subsequently by Elliott et al. (2007) and subsequently undertaken with patients with psychosis (Giersch et al., 2009; Schmidt et al., 2011; see Giersch et al., 2013; Elliott and Giersch, 2016, for reviews). The thresholds for perceived simultaneity recorded by Brecher were very reliably located at 53 ms and subsequently, for healthy control participants using a derivation of Brecher’s paradigm, were found by Elliott et al. (2007) to be located at 61 ms, 50–69 ms (Giersch et al., 2009), and 51 ms (Schmidt et al., 2011). Interestingly, the patients studies by Giersch et al. (2009) and Schmidt et al. (2011), revealed thresholds at close to double those of the healthy controls: in the case of Giersch et al. (2009), in one condition thresholds located at 50 ms in controls compare against those at 89 ms for patients with diagnosis of schizophrenia, while in two other conditions these values are 59 ms vs. 111 ms and 69 ms vs. 134 ms. For Schmidt et al. (2011), thresholds located at 51 ms for healthy controls compare against those located at 85 ms for patients with a first psychotic episode and 94 ms accompanying a diagnosis of schizophrenia. In all instances the threshold refers to the point at which perception switches from the perception of a simultaneity to the perception of succession and all tasks were completed successfully. Differences in the location of the threshold between patients and controls might be attributable to a change in the operational quantum, although this conclusion would require more substantive support before qualifying as a reliable scientific conclusion.

If the brain evolved its anatomical structure in order to allow neural activities to be performed with the greatest possible efficiency, it’s reasonable to assume that brain anatomy is a very general analog to the innumerable (but ordered) interactions between neurons engaged in cognition. And if interaction is important then of course brain anatomy will have evolved in a manner which ensures neural timing is subject to constraints (manifest in dynamical constants and time universals). All frontal cortex need do then, to surrogate for damage to other brain areas, is be able to articulate neurons within the same sets of temporal constraints that determine interactions within or between other cortical areas or structures. This seems plausible although of course the basic idea is almost certainly subject to more complex modifying constraints. In addition and to the best of our knowledge this hypothesis remains untested.

This argument suggests that error minimization is one of the major objectives of the efficient cognitive-neural system, but at what level should error be minimized, at the level of the process or at the level of the quantum determining the temporal operating characteristic of the process? This is a difficult question to answer in general, largely because there is little evidence of precisely how the brain functions, metrically, whilst performing cognitive tasks when damaged, or if obliged to reconfigure cognition over surrogate neural circuitry. Using an entrainment paradigm, Seifert et al. (2010) and Elliott et al. (2015) found blindsight as well as the temporary restoration of visual function within areas of the visual-blind field occurs following stimulation with stimuli flickering at very specific frequencies close to, or within the EEG gamma band. This research is unable to provide comparative measures of visual performance in the undamaged brain and so is unable to address the issue of error minimization by adjustment of process frequency. However and importantly, this research shows that by knowing which stimulus frequency promotes blindsight, also allows us to know the frequency able to partially reverse the effects of blindness.

## Conclusion

It makes sense to believe that regular, metrical properties of neural activity determine successful cognition and that cognition may recover from damage to the neural substrate by virtue of reconfiguration in surrogate neural substrate, to the extent that it is able to reconfigure itself in a regular and metrical pattern. However, showing this to be the case is an extremely difficult task, limited in no small way by the immense empirical task of building and verifying the necessarily metrical and dynamical models of successful cognitive function. Currently there are only two sets of relevant and necessarily systematic observations in this field: one of these reports the precise dynamical structure of the perceptual systems engaged in dynamical binding across form and time; the second, derived both empirically from perceptual performance data, as well as obtained from theoretical models, demonstrates a timing taxonomy related to a fundamental operator referred to as the time quantum. Their combination for a complete systematic examination depends upon a willingness to undertake lengthy experimental work of seemingly limited overall influence in terms of citations and publication impact. In addition, as every car mechanic knows, it is not possible to fit a new timing belt without lining up the timing marks and this requires an *a priori* understanding of the metrical properties of the system to be able to precisely evaluate hypotheses concerning the generalizability of taxonomic models of psychophysical and psychophysiological timing. The problem of a logical circularity may be avoided if research agendas employed theory such as Geissler’s TQM as a point of departure. Why is this important? In principle, an empirical understanding of timing in the dynamics of the cognitive microstructure is of enormous potential for targeted and precise treatment (such as that discussed above) for any abnormality arising at the brain systems level. At the present time our understanding of how to identify the timing marks are guided by TQM, but by very little other empirical work. Empirical support for TQM is confined to studies of apparent motion with a relationship to studies of oscillatory binding and although there is an, ‘in principle’ extension of precise metrical models of cognition to describe other types, including higher-order cognitive processes, generalization of TQM outside of perception remains hypothetical and, as such, a topic for future research. Nevertheless, the point of the current paper is to introduce a majorly under-represented view of cognition. This view possesses enormous scope for developing and refining our understanding of TQM, or a TQM-like model of dynamics in the cognitive microstructure. It is also importantly relevant to the exploitation of knowledge on precisely how the cognitive microstructure is organized dynamically. This might allow development of therapeutically oriented strategies that allow time and timing to repair impaired cognitive systems.

## Author Contributions

ME and NB are jointly responsible for the conception, drafting, and final approval of this contribution.

## Conflict of Interest Statement

The authors declare that the research was conducted in the absence of any commercial or financial relationships that could be construed as a potential conflict of interest.
